# Experience with comprehensive pharmacogenomic multi-gene panel in clinical practice: a retrospective single-center study

**DOI:** 10.3325/cmj.2022.63.257

**Published:** 2022-06

**Authors:** Vid Matišić, Petar Brlek, Vilim Molnar, Eduard Pavelić, Martin Čemerin, Kristijan Vrdoljak, Andrea Skelin, Damir Erceg, Davor Moravek, Ivana Erceg Ivkošić, Dragan Primorac

**Affiliations:** 1 St. Catherine Specialty Hospital, Zagreb, Croatia; 2 University of Zagreb School of Medicine, Zagreb, Croatia; 3 Genos Glycoscience Research Laboratory, Zagreb, Croatia; 4 Srebrnjak Children’s Hospital, Zagreb, Croatia; 5 Croatian Catholic University, Zagreb, Croatia; 6 University of Osijek Faculty of Dental Medicine & Health, Osijek, Croatia; 7 University of Split School of Medicine, Split, Croatia; 8 Eberly College of Science, State College, Penn State University, University Park, PA, USA; 9 The Henry C Lee College of Criminal Justice & Forensic Sciences, University of New Haven, West Haven, CT, USA; 10 University of Osijek School of Medicine, Osijek, Croatia; 11 University of Rijeka School of Medicine, Rijeka, Croatia; 12 Medical School REGIOMED, Coburg, Germany

## Abstract

**Aim:**

To assess the prevalence of actionable pharmacogenetic interventions in patients who underwent pharmacogenetic testing with a multi-gene panel.

**Methods:**

We retrospectively reviewed single-center electronic health records. A total of 319 patients were enrolled who underwent pharmacogenomic testing with the RightMed test panel using TaqMan quantitative real-time PCR method and copy number variation analysis to determine the SNPs in the 27 target genes.

**Results:**

Actionable drug-gene pairs were found in 235 (73.7%) patients. Relevant guidelines on genotype-based prescribing were available for 133 (56.7%) patients at the time of testing. Based on the patients’ genotype, 139 (43.6%) patients were using at least one drug with significant pharmacogenetic interactions.

**Conclusion:**

Two out of three patients had at least one drug-gene pair in their therapy. Further studies should assess the clinical effectiveness of integrating pharmacogenomic data into patients’ electronic health records.

The field of pharmacogenetics has been booming in the past decades, with research being focused on studying novel genetic variants that impact drug metabolism or pharmacological effect, which ultimately affects the patient’s response to a given dose of medication. Pharmacogenetics examines gene-drug interactions that change pharmacokinetic and/or pharmacodynamic properties of a drug (1). It is impossible to implement any of the principles of personalized medicine without determining the patients' pharmacogenetic profile before starting a new therapy (2).

Several professional organizations, namely, Clinical Pharmacogenetics Implementation Consortium (CPIC) and Dutch Pharmacogenetics Working Group (DPWG), provide comprehensive and understandable guidelines on genotype-based drug prescribing (3,4). Pharmacogenomic prescribing guidelines are growing in number and are available for various drug classes including the cardiovascular drugs, drugs affecting the central nervous system, gastrointestinal drugs, drugs that treat infectious and malignant diseases, immunosuppressives, analgesics, and other (5,6).

Several companies specialize in pharmacogenetic panels, making it easily accessible for patients and clinicians of various specialties to obtain the test results in a matter of days or weeks. These commercial tests are developed by industry stakeholders and can be implemented in various settings during the diagnostic or treatment process (7,8). They are comprehensive and include a number of genes that are important for the pharmacologic profile across drug groups, or targeted for a certain category of drugs, ie, psychiatric, analgesics, oncologic drugs, etc. Data on the rate of utilization and clinical utility of such tests are lacking. A recent study found that from 2013 until 2017 only 5712 insured US patients performed pharmacogenetic testing of at least one gene (9). The field of pharmacogenomics is still in its early stages. One of the principal problems is the education of health care providers responsible for ordering and interpreting the test results. In a recent survey, 84.3% of doctors from seven European countries deemed pharmacogenomics relevant for their practice, however 65.7% did not order a pharmacogenomic test in the last year (10). Potential implementation of pharmacogenomics in the clinical practice should be complemented with a clinical decision support tool integrated into the patient’s electronic health record (11,12). In Croatia, pharmacogenomic testing has been available for over a decade, with multiple studies examining population genetics and cost-effectiveness of pharmacogenomic guided therapies (13,14). However, commercial panel-based tests targeting multiple genes known to influence drug response is a new concept that was implemented in 2018 at St. Catherine Hospital in Zagreb (8,15,16).

The aim of this retrospective study was to assess the proportion of patients with actionable pharmacogenetic interventions in a single center from 2018 to 2021 who had undergone pharmacogenetic testing of 27 genes by using a commercial gene panel.

## Materials and methods

**Figure 1:**
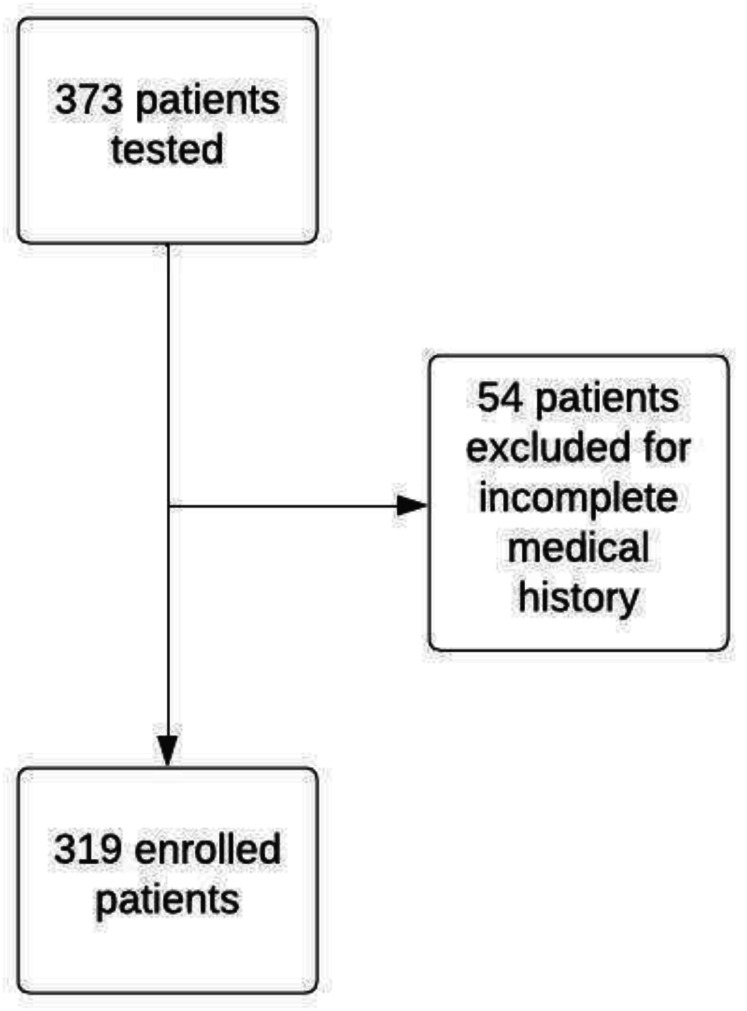
Patient enrollment flowchart.

St. Catherine Specialty Hospital's electronic medical records were retrospectively reviewed to extract the data on patients who had undergone pharmacogenetic testing with the RightMed test. From September 1, 2018, to December 10, 2021, 373 tests were performed. Complete medical history was missing for 54 patients, so 319 patients were enrolled (Figure 1).

The RightMed test was performed with buccal swabs, which were placed in a 1-mL saliva collection tube (RightMed test kit, ORACollectDx, DNA Genotek, Ottawa, ON, Canada). The DNA was analyzed by using the RightMedR panel with a TaqMan quantitative real-time PCR method and copy number variation analysis to determine the SNPs in the 27 targeted genes (Table 1). Genomic DNA was analyzed with PCR using Thermo Fisher TaqMan® (Thermo Fisher, Waltham, MS, USA) and/or LGC Biosearch BHQ® (Hoddesdon, UK) probe-based methods. Haplotypes, or combinations of inherited variants on a chromosome, were annotated according to legacy nomenclature for the genes. The test does not detect all known and unknown variations in the genes tested, nor does the absence of a detectable variant (designated as *1 for genes encoding drug metabolizing enzymes) rule out the presence of other, non-detected variants. These assays cannot differentiate between the maternal and paternal chromosomes. In the cases where observed variants are associated with more than one haplotype, OneOme infers and reports the most likely diplotype based on published allele frequency and/or ethnicity data. Results of the DNA analysis were then generated in individual test reports available on OneOme’s HIPAA-secure portal.

**Table 1:** Genes tested with the RightMed panel

Cytochrome P450 enzyme genes	*CYP1A2, CYP2B6, CYP2C9, CYP2C19, CYP2C cluster, CYP2D6, CYP3A4, CYP3A5, CYP4F2*
Second phase of drug metabolism enzyme genes	*TPMT, UGT1A1*
Other enzymes responsible for drug metabolism genes	*DPYD, VKORC1, NUDT15*
Drug transporter genes	*SLC6A4, SLCO1B1*
Drug receptor genes	*HTR2A, HTR2C, DRD2A, OPRM, COMT, GRIK4*
Other proteins responsible for drug metabolism and safety genes	*IFNL4, HLA-A, HLA-B, F II, F V*

Complete medical history from the 319 enrolled patients was analyzed to find the data on the number of drugs in patients’ therapy. Patients were subcategorized into three groups: those who used up to 3 drugs (group 1), those who used between 4 and 8 drugs (group 2), and those who used 9 and more drugs (group 3).

Pharmacogenomic test reports were analyzed according to the drugs the patient reported using. We assessed the number of drugs with known gene-drug interactions, the number of drugs with available genotype-based guidelines, and the number of drugs with significant gene-drug interactions according to the patients’ phenotype. The latter were then categorized with respect to major drug groups.

### Statistical analysis

We used descriptive statistics to summarize genotype and phenotype data (drug metabolizer rates). Normality of the distribution was tested with the Kolmogorov-Smirnov test. The Kruskal-Wallis and Mann-Whitney test were used to assess the differences between the groups. Statistical analysis was performed with SPSS 23.0 (IBM, Armonk, NY, USA), with the significance level set at *P* < 0.05.

## Results

The samples were collected from 140 men and 179 women. The median age was 54 years (range 1-88 years). Women were significantly older than men (57 years vs 51 years; Mann-Whitney, *P* = 0.019).

Out of 319 patients, 155 had up to 3 drugs in their therapy (group 1), 126 had between 4 and 8 drugs in therapy (group 2), while 38 patients had 9 or more drugs in their therapy (group 3). Group 1 patients were significantly younger than group 2 (Mann-Whitney test, *P* < 0.001) and group 3 patients (Mann-Whitney test, *P* < 0.001). Forty-six patients had no drugs in their therapy.

When observing the complete data set, only 84 patients had no interactions between drugs in their therapy, while 235 (73.7%) patients had at least one gene-drug pair. Out of these 235 patients, 111 (34.8%) patients had 3 or more gene-drug pairs. For drugs in the therapy of 133 (56.7%) patients, there were available genotype-based prescribing guidelines, with 95 (40.4%) patients having at least one such drug in their therapy, 30 (12.8%) having two, 6 (2.6%) having three, and 2 (0.9%) having four.

The pharmacogenetic analysis demonstrated that 139 (43.6%) patients had significant gene-drug interactions to at least one drug present in therapy, while 180 (56.4%) patients did not have significant gene-drug interactions. With respect to the therapeutic groups of drugs that were found to have significant gene-drug interactions, the highest frequency was observed for psychiatric drugs (47.6%), followed by gastroenterology drugs (25.5%), and cardiovascular drugs (18.6%) (Table 2).

**Table 2:** Detected drugs with significant gene-drug interactions in patients’ therapy at the time of testing

Drug group	Number of patients	Drugs (number of patients)
psychiatric	69	diazepam (36)
		olanzapin (10)
		clozapine (8)
		sertraline (8)
		risperidon (7)
		fluvoxamin (6)
		paroxetin (6)
		aripiprazol (5)
		escitalopram (4)
		haloperidol (3)
		alprazolam (2)
		mirtazapin (2)
		promazin (2)
		venlafaxine (2)
		bupropion (1)
		clomipramine (1)
		fluoxetine (1)
gastroenterological	37	pantoprazol (29)
		esomerazol (5)
		rebeprazol (5)
		rebeprazol (1)
		lansoprazol (1)
cardiovascular	27	metoprolol (10)
		atorvastatin (8)
		losartan (3)
		carvedilol (3)
		propafenone (2)
		simvastatin (2)
		amlodipine (2)
		timolol (2)
		felodipine (1)
		eplerenon (1)
anti-inflammatory	14	diclofenac (7)
		ibuprofen (6)
		celecoxib (2)
		flubiprofen (1)
analgesics/anesthesiological	13	tramadol (9)
		codeine (1)
		oxycodone (1)
		lidocaine (1)
		morphine (1)
anticoagulant/antiplatelet	11	clopidogrel (7)
		warfarin (4)
		ticagrelor (1)
neurological	8	lamotrigine (3)
		caffeine (1)
		phenytoin (1)
		rasagilin (1)
		clobazam (1)
hematological/oncological	4	methotrexate (2)
		tamoxifen (1)
		dasatinib (1)
urological	3	tamsulozine (3)
sleep medicines	2	zolpidem (2)
used to treat infectious diseases	2	claritromycine (1)
		peginterferon 2A (1)
		peginterferon 2B (1)
allergological/pulmonary	2	loratadine (2)
endocrinological	1	glimepiride (1)
rheumatological	0	/
used to treat genetic diseases	0	/
immunosuppressives	0	/

**Figure 2:**
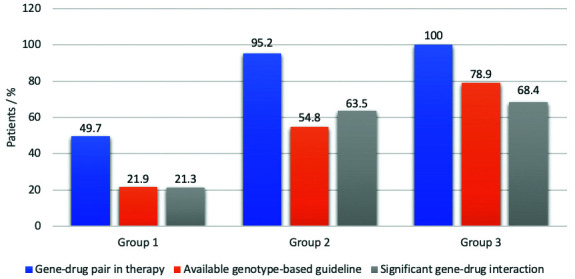
Subgroup analysis with respect to the number of drugs in patients’ therapy.

A subgroup analysis was performed with respect to the number of drugs in patients’ therapy (Figure 2). In group 1 (n = 155), 77 (49.7%) patients had at least one gene-drug pair in their therapy. A genotype-based guideline was available for 34 (21.9%), and 33 (21.3%) patients had at least one actionable gene-drug interaction. In group 2 (n = 126), 120 (95.2%) patients had at least one actionable gene-drug pair. A genotype-based guideline was available for 69 (54.8%) patients, and 80 (63.5%) patients had at least one actionable gene-drug interaction. In group 3 (n = 38), all of the patients had at least one gene-drug interaction in their therapy. A genotype-based guideline was available for 30 (78.9%) patients, and 26 (68.4%) patients had at least one actionable gene-drug interaction.

## Discussion

The majority of our patients (73.7%) in our study had at least one gene-drug pair known to affect the pharmacologic properties of the drug, which indicates a growing need for the implementation of pharmacogenomics in patient care. The proportion of patients with gene-drug interactions increased with respect to the number of drugs in their therapy. This high percentage was reported by other groups as well. Elchynski et al reported this rate to be between 70% and 75% for patients who were genotyped for *CYP2C19* and *CYP2D6* (17). Overall, 46 (14.4%) patients in our study underwent pharmacogenomic testing preemptively, before using any drugs. Preemptive testing is already advocated as the best strategy to maximally utilize the pharmacogenomic tests (18). Prospective studies confirmed that this approach could be useful in the primary care setting. Van der Wouden et al reported that 24.2% of newly prescribed medications were actionable and required therapeutic adjustment in 2 years in the primary care setting (19). Similarly, Youssef et al reported this percentage to range between 19.1% and 21.1% (20). Bank et al reported it to be 23.6% (21). In our study, significant gene-drug pairs that can alter the pharmacologic effect of drugs were present in 139 (43.6%) patients.

Due to the retrospective nature of the study and no available data on therapeutic intervention made after the test results were obtained, we cannot assess the prospective clinical utility of the performed pharmacogenomic tests and benefit to the patients. Furthermore, the presence of actionable interventions when found retrospectively should not be taken as an imperative to change a previously well-established therapy. Pharmacogenomic test results should be interpreted by professionals trained in the field who understand that the reasons for the history of adverse drug reactions or the lack of therapeutic effect do not necessarily include only the patient's genotype, but also the epigenetic changes, comorbidities, age, and other factors (15,22). As demonstrated by our results, younger patients used less drugs in their therapy compared with older patients. Older patients in general are at a greater risk for adverse drug reactions (ADRs) because of the metabolic changes associated with aging. When the number of drugs in their therapy increases, the risk of ADRs increases exponentially, which may lead to decreased compliance, poor quality of life, and unnecessary drug expenses (23). We observed that the percentage of significant gene drug interactions was higher with respect to the number of drugs in the patients’ therapy. Therefore, we presume that pharmacogenomic testing for this group of patients could improve their therapeutic outcome and reduce the number of experienced ADRs. However, further research assessing the therapeutic intervention based on pharmacogenomic testing, with a longer follow-up, is warranted to fortify the rationale for testing in routine clinical practice.

Psychiatric drugs are the archetypal drugs heavily influenced by gene-drug interactions resulting in adverse drug reactions and the lack of therapeutic effect. This often leads to a trial-and-error approach to prescribing. In this study, we found significant gene-drug interactions for clozapine in 8 patients. Clozapine pharmacokinetics is influenced by *CYP3A4* and *CYP3A5* activity, as well as polymorphisms in *HLA-B* that can cause clozapine-induced neutropenia (24). Information provided by pharmacogenetic testing should be used to personalize treatment in psychiatry. This is best done by combining genotyping with therapeutic drug monitoring (25). A meta-analysis including 1556 patients showed that the outcomes of major depressive disorder treatment were superior in patients who received genotype-guided treatment (26). Our results support the potential of proactive pharmacogenomic testing for patients with psychiatric conditions, as the majority of detected actionable gene-drug interactions were detected for these drugs. Furthermore, the International Society of Psychiatric Genetics decided in favor of testing for polymorphisms in *CYP2C9*, *CYP2D6, HLA-A,* and *HLA-B* at the current level of understanding, while also indicating the potential of a wider implementation of pharmacogenomic testing in psychiatry (27).

In our study, proton pump inhibitors (PPIs) were the second most common drug group with significant gene-drug interactions. PPIs' therapeutic effect is partly determined by polymorphisms in the *CYP2C19* (28). The favorable side-effect profile of these commonly prescribed drugs allows them to be implemented in the case of reduced metabolism. However, patients who are rapid metabolizers are at greater risk for the lack of therapeutic effect (29). Therefore, it would be prudent to determine these polymorphisms in cases of a lack of therapeutic effect.

The third most common group of drugs with a significant gene-drug interactions in our patients was the cardiovascular drugs. Statins and beta-blockers are among the most commonly used drugs. Atorvastatin and metoprolol particularly (which were the two most common gene-drug pairs with significant interactions in the cardiovascular group in this population) are among the top 10 most prescribed drugs in the United States (30). In Croatia, atorvastatin was the third most consumed drug in 2020 (31). Statins and beta-blockers also have a well-established gene-drug interaction profile with genotype-guided therapy guidelines for statins already available to the attending clinician (32). A recent randomized controlled trial demonstrated that the adherence to this guideline did not reduce the effect of statin therapy (33). Another category of drugs closely related to cardiovascular disease are anti-platelet and anti-coagulation drugs, which were actionable in 11 patients. Clopidogrel was actionable in 7 patients. Patients who have decreased *CYP2C19* activity using clopidogrel have an increased risk of ischemic events due to the lack of effectiveness of secondary prevention (34). Recently, it was found that a genotype-guided strategy was non-inferior to standard practice, but with a lower incidence of bleeding events (35).

Anti-inflammatory and analgesic drug-gene pairs were found in 14 and 13 patients’ therapy regiments, respectively. The effect of genetic variance on their pharmacological effect and side-effect profile is well-established (36,37). Genotype-based guidelines are available for some of the most prescribed drugs including ibuprofen, meloxicam, celecoxib, and others, allowing for their more precise prescription to avoid the side effects of their decreased metabolism (38). A genotype-based guideline is available for tramadol, codeine, and hydrocodone dosing based on *CYP2D6* genotype (39). Opioid analgesics are also known for their addictive potential, with genetic factors possibly having an important role in this mechanism. Currently, predictive models are being developed to help determine the risk of addiction (40).

In order to reap clear benefits for the patient, pharmacogenetic test results should be integrated into the patient’s electronic health record, where they can be correlated with other important patient-related factors that could also affect the therapeutic outcome. The integration of these data should foremost be used to avoid preventable, genotype-determined, severe adverse drug reactions and to identify the patients who are at risk of a reduced therapeutic outcome (particularly important in secondary prevention) (12). Such integration enabled 97% of patients to reuse the pharmacogenomic test results in the future (19). This can be further reinforced with the development of more genotype-based prescribing guidelines, which, when integrated, can serve as an easily accessible reference. The current study showed that for 56.7% of the patients who had at least one gene-drug pair, there was an established prescribing guideline. In the future, with more research being available, this proportion is likely to increase.

The limitations of this study include its retrospective design, which did not allow for an insight into the change of patients’ therapy after genotyping. Furthermore, at the time of testing the patients’ reports might not have included some more commonly prescribed over-the-counter drugs with known gene-drug interactions, ie, ibuprofen, diclofenac, etc.

The results of this study demonstrate that multi-gene pharmacogenomic testing provides actionable clinical information in two out of three patients. The increasing digitalization of health care in developed countries provides the necessary infrastructure for a successful implementation of proactive pharmacogenomic testing, which could enable personalized therapy prescribing. However, further effort has to be made to educate the patients and health care providers on the benefits of personalized prescribing.
